# A predictive nondestructive model for the covariation of tree height, diameter, and stem volume scaling relationships

**DOI:** 10.1038/srep31008

**Published:** 2016-08-24

**Authors:** Zhongrui Zhang, Quanlin Zhong, Karl J. Niklas, Liang Cai, Yusheng Yang, Dongliang Cheng

**Affiliations:** 1 Fujian Provincial Key Laboratory of Plant Ecophysiology, Fujian Normal University, Fuzhou, Fujian Province 350007, China; 2 Key Laboratory of Humid Subtropical Eco-geographical Process, Ministry of Education, Fuzhou, Fujian Province 350007, China; 3 Plant Biology Section, School of Integrative Plant Biology, Cornell University, Ithaca, NY 14853, USA; 4 College of Information Engineering, Jiangxi University of Technology, Nanchang, Jiangxi Province 330098, China

## Abstract

Metabolic scaling theory (MST) posits that the scaling exponents among plant height *H*, diameter *D,* and biomass *M* will covary across phyletically diverse species. However, the relationships between scaling exponents and normalization constants remain unclear. Therefore, we developed a predictive model for the covariation of *H*, *D*, and stem volume *V* scaling relationships and used data from Chinese fir (*Cunninghamia lanceolata*) in Jiangxi province, China to test it. As predicted by the model and supported by the data, normalization constants are positively correlated with their associated scaling exponents for *D* vs. *V* and *H* vs. *V*, whereas normalization constants are negatively correlated with the scaling exponents of *H* vs. *D.* The prediction model also yielded reliable estimations of *V* (mean absolute percentage error = 10.5 ± 0.32 SE across 12 model calibrated sites). These results (1) support a totally new covariation scaling model, (2) indicate that differences in stem volume scaling relationships at the intra-specific level are driven by anatomical or ecophysiological responses to site quality and/or management practices, and (3) provide an accurate non-destructive method for predicting Chinese fir stem volume.

The accurate estimation of standing plant biomass is essential for understanding and predicting the effects of forest ecosystem processes (e.g. energy, nutrient, water, and carbon fluxes) on regional and global carbon cycles^1^,^2^,^3^,^4^. A convenient and widely used method for biomass estimation is provided by equations that interrelate plant biomass (*M*) and stem/trunk diameter (*D*) that take the form *M* = β*D*^α^, where β is a normalization constant and α is the scaling exponent^5^,^6^,^7^,^8^. Since the numerical values of β and α can differ among species, stand age, site characteristics, climate, and stand density^5^,^6^,^7^,^9^,^10^, they are typically estimated via regression of log-transformed data for *D* and *M* data obtained from destructive sampling methods. This approach is time consuming and expensive, and thus generally restricts data collections to small areas, plant sizes, and sample numbers. More efficient and economical methods for estimating allometric parameters would help considerably.

In addition to empirical model fitting approaches, theoretical models have also been used to predict and estimate allometric scaling exponents. For example, metabolic scaling theory (MST)^11^,^12^,^13^,^14^,^15^ hypothesizes that evolutionary optimization of vascular transport hydraulics has resulted in plant metabolic rates that scale as 3/4 power of *M* and that *M* scales as the 8/3 power of *D*. Several authors have used these and other MST predictions to develop allometric models for interrelating *M* and *D*^4^,^6^,^9^,^16^,^17^ and to evaluate the generality of predicted or estimated allometric parameters, although other authors have concluded that the power-law scaling exponents vary considerably within and across taxa^6^. Additionally, several studies have argued that MST as originally formulated^11^,^12^ cannot explicitly account for the range and origin of variation of plant metabolic scaling exponents^18^,^19^,^20^,^21^. For example, mass-scaling exponents of metabolic rates are close to unity for saplings (isometry) and decrease as trees grow in size^22^,^23^,^24^, implying that metabolic scaling relationships vary though ontogeny. To address the concerns, Niklas and Enquist^24^ and Enquist *et al*.^25^ used biomechanical and space-filling arguments to suggest how metabolic scaling of seedlings and saplings should deviate from the original MST predictions^11^. Further, Niklas^26^ demonstrated that the scaling of height with respect to diameter decreases from nearly isometric (for small and juvenile trees) to a 2/3 power for older more mature trees. Niklas and Spatz^22^ also developed a hydraulic model that predicts a log-log nonlinear *H* (and *M*) vs. *D* relationship that predicts a shift from an isometric to an allometric scaling exponent across species and habitats.

To address these issues and to expand their theoretical underpinnings, Price *et al*.^27^ extended MST to show that the biomass scaling exponents relating *M*, *H*, and *D* covary, a feature that was already demonstrated by Niklas and Spatz^22^ and Sileshi^28^. Consequently, the scaling exponents of *M* vs. *D* can be estimated from those of *H* vs. *D*, which provides an attractive method for estimating tree biomass scaling relationships because data for *D* and *H* can be collected non-destructively. Indeed, if tree trunks can be modeled as simple cylindrical or truncated conical geometries, tree biomass can be related to diameter and height as *M* ∝ *D*^2^*H*^9^. Furthermore, if the scaling exponent relating height and stem diameter is denoted as α_1_, we see that 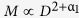, which is a more general expression of the *M*, *D*, and *H* scaling interrelationship.

However, although biomass-scaling relationships clearly involve covariation among the scaling exponents for the relationships among *M*, *D*, and *H* (see details in Materials and Methods), the extent to which these relationships mediate the numerical values of normalization constants (i.e., β-values) remains unclear. Prior studies have shown that scaling exponents are inversely related with their corresponding scaling constants in *M* vs. *D* relationships^6^,^10^,^28^. For example, using a collection of 223 allometric equations relating biomass to diameter, Zianis and Mencuccini^6^ have shown that normalization constants are negatively correlated with the scaling exponents governing *M* vs. *D* scaling relationships. If this relationship holds true generally, a “prediction model” for estimating tree biomass can be established by recasting biomass-scaling relationships in terms of an inverse relationship between the numerical values of scaling exponents and normalization constants. Specifically, the scaling of *M* with respect to *D* can be estimated from the scaling of *H* with *D*, and the normalization constants can be estimated using the specific function between scaling exponents and constants as suggested by Zianis and Mencuccini^6^.

Nevertheless, empirical testing of the biomass scaling relationships proposed by Price *et al*.^28^ has been based primarily on the datasets collected from different species and biomes. More experimental work is needed to gain insight into the mechanisms of covariation among scaling exponents and normalization constants at the level of individual species. Since plant functional traits can influence metabolic scaling relationships^29^,^30^,^31^, predictions for the covariation of scaling relationships among *D*, *H* and *V* must be tested at the intra-specific level because of species-specific differences among species (e.g. wood density). Under any circumstances, it is necessary to verify whether the covariation between the numerical values of scaling exponents and normalization constants hold true at the intra-specific level.

In light of the theoretical and practical importance of understanding the mathematical and biological relationships among scaling exponents and their corresponding normalization constants, we used the stem volume data of *Cunninghamia lanceolata* (Lamb.) Hook. (Chinese fir) in Jiangxi province, China, to (1) examine the variations of the scaling relationships between stem volume, diameter, and height, (2) test whether an inverse relationship between scaling exponents and related constants holds true, (3) verify whether the covariation of scaling exponents in these stem relationships support the mathematical functions proposed by Price *et al*.^27^, and (4) test whether the prediction model emerging from our approach successfully estimates stem volume.

## Materials and Methods

### Species and Study Area Selection

*Cunninghamia lanceolata* (Lamb.) Hook. (Chinese fir) is an evergreen conifer in the *Taxodiaceae* (Redwood) family. This species was selected because it is one of the most important commercial trees in China^32^ and because it is grown in a variety of sites. In the first half of 1988 and 1999, twenty-four sites in the Jiangxi Province were selected to investigate tree stem (trunk) growth (Supplementary Fig. S1). The original planting density was 3300 trees·ha^−1^. For the first three years, the forest was tended twice every year, after which it was left undisturbed for the duration of the experiment. Thinning operations were conducted using chain saws and heavy equipment after 7–10 years of the initial planting of trees. About 30% trees in the plantation were felled. When necessary, a second thinning operation was conducted to maintain an appropriate space between neighboring trees after 12–15 years of the initial plant of trees, the average reserve density is about 1800 trees·ha^−1^. All of the sites were located in subtropical monsoon climatic regions. Mean annual temperature ranged from 16.5 °C to 19.5 °C and mean annual precipitation from 1421 mm to 1962 mm (Supplementary Table S1).

### Field Measurements

Because *C. lanceolata* is the main forestation species in Jiangxi province, all trees were collected from plantation sites. Circular forest research plots were established with areas of 600 m^2^. Because prior work had shown that the architecture of the forest canopy is an important determinant of the scaling relationship between tree height and diameter^33^, efforts were made to eliminate differences among canopy densities by drawing data only from sampling plots where the vertical projection of forest crowns was over 60%. This protocol identified 24 sites that could be sampled.

At each site, individuals spanning a wide range of sizes were selected in order to properly characterize the size distribution of the local stand (Supplementary Table S1). Because the number of plots varied across sites, the number of sampled individuals ranged between 6 to 185 (Supplementary Table S2).

Data were obtained by first measuring trunk diameter at breast height (DBH; 1.3 m from ground-level). Trees were then felled using a chain saw, and total height was measured using a steel tape. Stem discs were then taken at 1.3 m above the base and every 1 m for *H* < 10 m or 2 m for *H* ≥ 10 m thereafter. An additional disc was taken at 0.5 m above the base for trees <10 m. Finally, the stem volume of each trunk section was calculated based on the geometric shape of the segments. For example, the stem volume for the top section (above the last sample disc) was calculated using the formula for a truncated cone. Total trunk volume was calculated subsequently as the sum of all trunk sectional volumes.

### Statistical protocols

Data for *D, H* and *V* from each of the 24 sites were log_10_-transformed. Because functional rather than predictive relationships were sought, reduced major axis (RMA) regression was used to determine the scaling exponents (α) and normalization constants (log β) for log–log linear regression curves (see Supplementary Table S2). The parameter φ (see Supplementary Eqs (6–8)) was calculated using nonlinear regression analyses in SPSS Statistics 17.

Because trees were required to determine the numerical values required to develop a prediction model and because trees were aslo necessary to test the prediction model, 12 of the 24 sites (from Anfu to Ruichang, listed in Supplementary Table S1) were used to establish the prediction model and the remaining 12 sites (from Ruijin to Yongxin; see Supplementary Table S1) were used for testing the model. Specifically, the numerical values of the scaling exponents and constants of *V* vs. *D* of 12 sites were used to estimate the parameters *c* and *d* in Supplementary Eq. (10). Then, using the estimated parameter φ, a site-specific stem volume prediction model was developed and used to predict stem volume based on measurements of *D* and the associated scaling exponents of *H* vs. *D* for the second set of 12 sites. It must be noted that ordinary least squares (OLS) regression analyses were used to establish the prediction model (Supplementary Eq. (11)) because the objective was to predict standing biomass by means of predicting stem volume.

RMA and OLS regression analyses were performed using the Standardized Major Axis Tests and Routines (SMATR) software package^34^,^35^. The software package SMATR was also used to determine whether the numerical values of scaling exponents differed among the 24 sites, which can provide the Model Type II equivalent of OLS standard analyses of covariance (ANCOVA). The significance level for testing scaling exponent heterogeneity was *P* < 0.05 (i.e. slope heterogeneity was rejected if *P* > 0.05).

The reliability of using this approach to predict stem volume was assessed numerically by calculating the mean absolute percentage error (MAPE) as suggested by Sileshi^27^ using the formula: 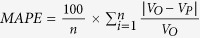, where *V*_*O*_ and *V*_*P*_ denote the observed and predicted stem volume, respectively.

## Results

### Volume-scaling relationships

**Figure 1:**
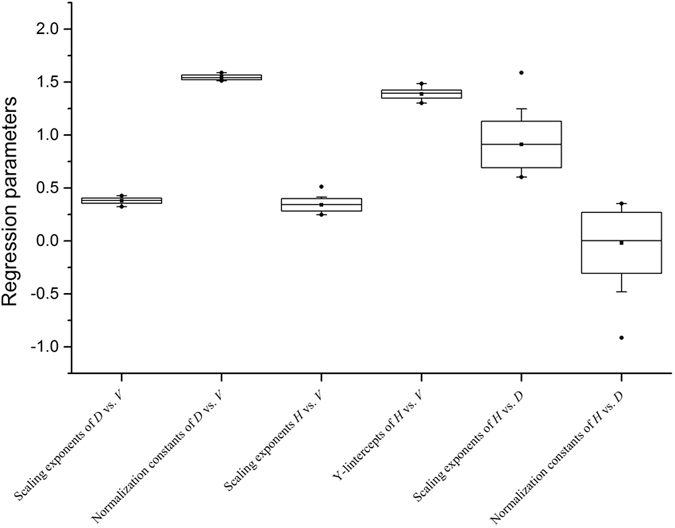
The regression parameters (scaling exponents and normalization constants, respectively) for volume scaling relationships of height *H*, diameter *D*, and stem volume *V* for *Cunninghamia lanceolata* at 24 sites in Jiangxi Province. The numerical values are shown in Supplementary Table S1.

The numerical values of the scaling exponents for volumetric scaling were significantly heterogeneous across sites (*P* < 0.005) (Supplementary Table S2). The mean scaling exponent of all sites was 0.380, with the smallest scaling exponent at Guixi and the largest at Anfu (i.e. α = 0.323 and 0.427, respectively). For all sites, *D* scaled as 0.386 power of *V* (95% CIs = 0.383–0.390, *n* = 1273, *r*^2^ = 0.969). Likewise, the scaling exponents relating height and volume varied significantly among sites (*P* < 0.005; Supplementary Table S2), and ranged from 0.323 to 0.427, with a mean of 0.341 (Fig. 1). Pooling all of the data gave α = 0.331 and log β = 1.389 (*n* = 1273, *r*^2^ = 0.870). The scaling exponents for the *H* vs*. D* relationship differed significantly among sites (*P* < 0.005), and ranged from 0.603 to 1.589 (Supplementary Table S1).

### The allometric covariation of volume-scaling relationships

#### The covariation of scaling exponents

**Figure 2:**
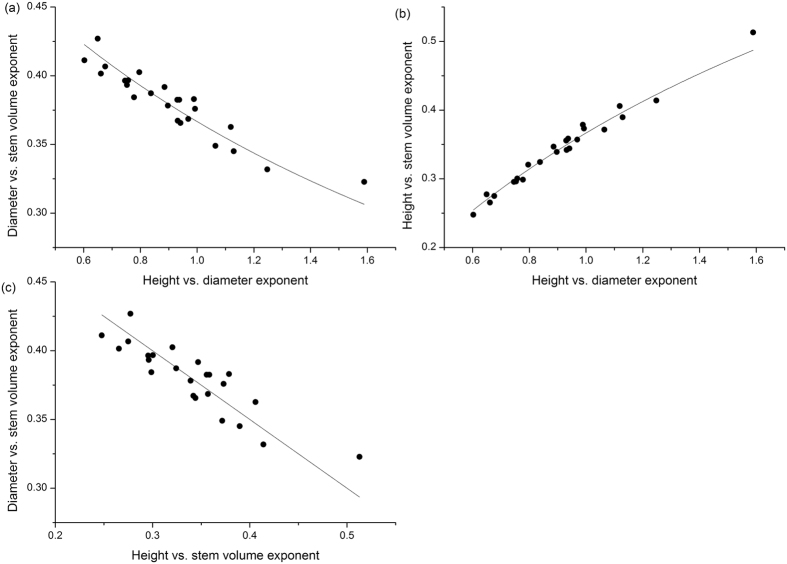
Allometric covariation of volume scaling exponents estimated using RMA regressions of 24 sites in Jiangxi Province. (**a**) Relationship between scaling exponents for *D* vs. *V* and *H* vs. *D*. Axes represent the function *y* = φ/(2 + *x*), where φ is 1.10 (95% CI = 1.09–1.11, *r*^2^ = 0.867). (**b**) Relationship between scaling exponents for *H* vs. *V* and *H* vs. *D*. Axes represent the function *y* = φ/(1 + 2/*x*), where φ is 1.10 (95% CI = 1.09–1.12, *r*^2^ = 0.975). (**c**) Relationship between scaling exponents for *D* vs. *V* and *H* vs. *V*. Axes represent the function *y* = (φ − *x*)/2, where φ is 1.10 (95% CI = 1.09–1.11, *r*^2^ = 0.703).

The empirical data agreed well with the predicted covariations of stem volume scaling relationships (Fig. 2). Specifically, the observed relationships between the scaling exponents for *D* vs. *V *^(*y*′)^ and *H* vs. D^(*x*)^ closely followed the predicted function of Supplementary Eq. (8) (i.e. 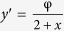), with *φ* = 1.10 (95% CIs = 1.09–1.11, *r*^2^ = 0.867) (Fig. 2a). The relationship between scaling exponents for *H* vs. *V*^(*z*′)^ and *H* vs. *D*^(*x*)^ was governed by the function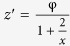, where φ is 1.10 (95% CI = 1.09–1.12, *r*^2^ = 0.975) (Fig. 2b). The observed relationships between the scaling exponents for *D* vs. *V* and *H* vs. *V* also complied with the predicted function 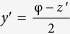, with *φ* = 1.10 (95% CIs = 1.09–1.11, *r*^2^ = 0.703) (Fig. 2c). Importantly, the *φ*–value calculated for the three covariation curves had the same identical value of 1.10 (Fig. 2).

#### The covariation of scaling exponents and constants

**Figure 3:**
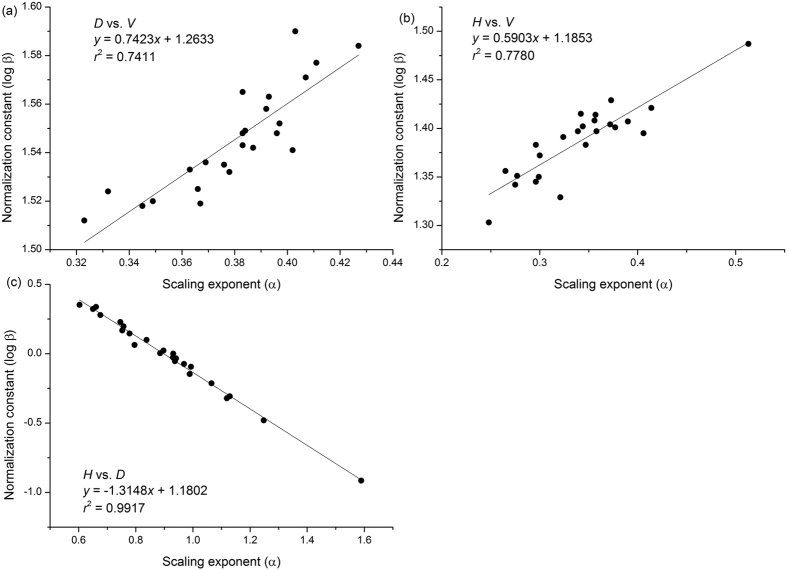
Normalization constants versus scaling exponents using reduced major axis (RMA) regression for *C. lanceolata* at 24 sites. (**a**) Relationship between *D* vs. *V*. (**b**) Relationship between *H* vs. *V*. (**c**) Relationship between *H* vs. *D.*

The scaling exponents and normalization constants were statistically significantly correlated with one another (Fig. 3). Specifically, normalization constants positively correlated with the scaling exponents for *D* vs. *V* (i.e. *y* = 0.74*x* + 1.26, *n* = 24, *r*^2^ = 0.741, *P* < 0.01) (Fig. 3a) and *H* vs. *V* (i.e. *y* = 0.59*x* + 1.19, *n* = 24, *r*^2^ = 0.778, *P* < 0.01) (Fig. 3b). In contrast, the relationship between the constants and the exponents for *H* vs. *D* was significantly negative (i.e. *y* = −1.31*x* + 1.18, *n* = 24, *r*^2^ = 0.992, *P* < 0.01) (Fig. 3c).

**Figure 4:**
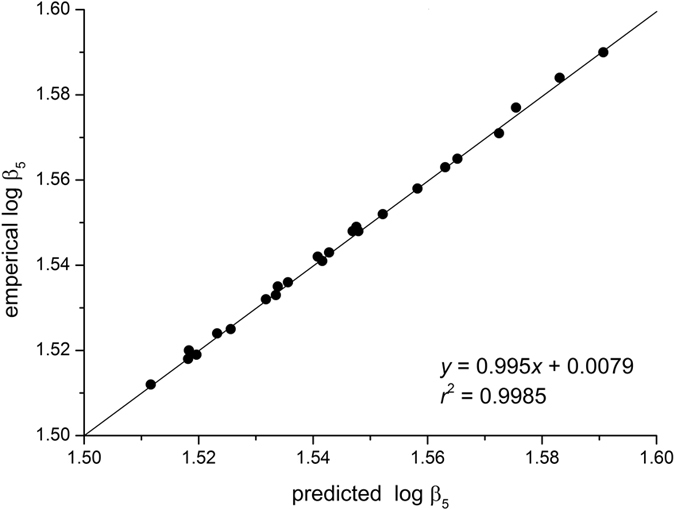
Relationship between empirical normalization constants (β_5_) of reduced major axis (RMA) regression for *D* vs. *V* at 24 sites and the predicted normalization constants based on the formula 
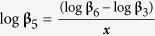
, where log β_6_ is the normalization constant for *H* vs. *V*, log β_3_ and *x* are the normalization constant and scaling exponent for *H* vs. *D*, respectively. The dashed line is isometric with a slope of one.

Furthermore, as predicted by Supplementary Eq. (9), the *H*-*D* scaling exponents were significantly correlated with the covariation of normalization constants in the stem volume scaling relationship (i.e. *y* = 0.995*x* + 0.0079, *n* = 24, *r*^2^ = 0.999) (Fig. 4).

#### Predictions and there percent prediction errors

**Figure 5:**
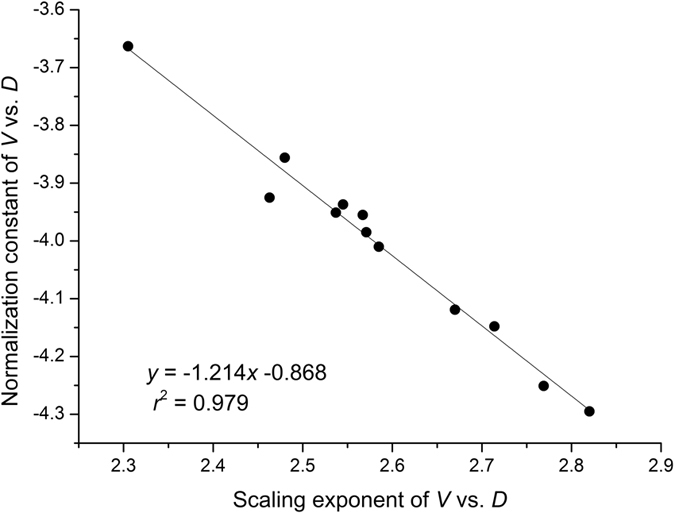
Relationship between normalization constants and scaling exponents using the OLS regression of *V* vs. *D* for *C. lanceolata* at 12 model development sites in Supplementary Table S1. Normalization constants and exponents were calculated using ordinary least squares regression. Solid line is OLS regression line (*r*^2^ = 0.979).

A significant negative relationship was observed between empirically determined normalization constants (i.e. log β) and the *V* vs. *D* scaling exponents (i.e., 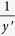) across the 12 sites (for details, see Material and methods and Supplementary Table S1) used to develop the prediction model, i.e.


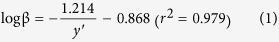


when *V* is expressed in m^3^ and *D* in cm (Fig. 5).

The empirically determined scaling exponents of *H* vs. *D* were applied to Supplementary Eq. (8) to estimate 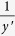. These values were then applied to Supplementary Eq. (12) to calculate the corresponding normalization constants for each model calibrated site (Supplementary Table S1). Lastly, a non-destructive model for estimating the stem volume of *C. lanceolata* was obtained, i.e.,


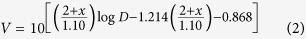


where *x* is the empirically determined site-specific scaling exponent for *H* vs. *D*.

**Figure 6:**
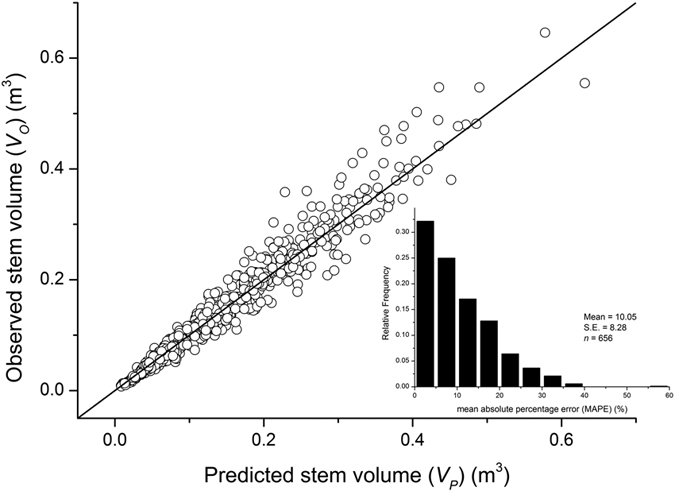
Relationship between observed and predicted stem volume of *Cunninghamia lanceolata* across the second set of 12 model calibrated sites listed in Supplementary Table S1. The insert plot is frequency distributions of the mean absolute percentage error (MAPE) based on the Eqn (13). The mean and S.E. values are presented.

Across all of the model calibrated sites, the mean absolute percentage error (MAPE) was 10.50 ± 0.32 SE, and 57% of all trees had MAPE values less than 10% (Fig. 6).

## Discussion

### The variation in volume-scaling relationships

A number of previous studies have predicted heterogeneity in the numerical values of the scaling exponents of stem volume scaling relationships. For example, three biomechanical models have been proposed to explain the scaling of *H* with respect to *D*. These are the geometric similarity model, which assumes height will scale isometrically with respect to diameter (*H* ∝ *D*^1/1^), the elastic similarity model, which assumes height will scale as the 2/3 power of diameter (i.e. *H* ∝ *D*^2/3^), and the constant stress similarity model, which assumes height will scale as the 1/2 power of diameter (i.e. *H* ∝ *D*^1/2^)^36^,^37^. Our data demonstrate that none of these models can be applied to our experimental system because significant variation in the numerical values of the scaling exponents of the stem volume scaling relationships exist for *C. lanceolata* (Supplementary Table S2; Fig. S2). Specifically, for the scaling relationship of *H* vs. *D*, five sites had scaling exponents with 95% CIs that included 2/3, twelve sites included 1.0, one site included both 2/3 and 1.0, and five sites included neither 2/3 nor 1.0. Further, across all of the sites, height scaled as the 0.86 power of diameter (95% CI = 0.84–0.88) (Supplementary Table S2). These results diverge significantly from all of the aforementioned models, indicating that no single optimal scaling exponent exists for Chinese fir. Indeed, many studies have demonstrated that tree scaling relationships for height, diameter and biomass are variable rather than constant^27^,^38^,^39^,^40^,^41^,^42^,^43^.

In addition to empirical studies, using a growth-hydraulic model, Niklas and Spatz^21^ predicted a nonlinear (convex) relationship between height and diameter through tree ontogeny, which agrees with empirical observations^26^ and implies that scaling relations will vary among trees of different sizes^16^. Similarly, Enquist *et al*.^25^ have shown a curvilinear relationship between tree height and diameter and a similar scaling transition for metabolism and biomass. Given that our results indicate that biomass scales nearly as the 1.10 power of stem volume (Fig. 2), the observed variation in scaling relationships of Chinese fir might, at least in part, reflect differences in scaling relationships between trees of different ontogenetic stages driven ultimately by anatomical or ecophysiological responses to site quality and/or management practices.

### The covariation of volume scaling relationships

It has long been recognized that size-dependent variation in *H* vs. *D* scaling relationships can be important in shaping other allometric relationships^44^. For instance, Dai *et al*.^45^ demonstrated that the scaling exponent of height with respect to diameter decreases with increasing drought stress such that, for a given diameter, drought stressed trees are proportionately shorter, leading to a systematic change in the plant density–mass relationship. Our data demonstrate that the scaling exponents of the stem volume scaling relationships covary and that the changes agree with the equations derived from the biomass scaling relationships suggested by Price *et al*.^27^ (see Supplementary Eq. (8), Fig. 2), which indicates that the intraspecific covariation in scaling exponents for Chinese fir plantations across different locations holds true as well as across species. Thus, our results indirectly support the hypothesis that plant growth can adjust network geometry and hydraulic function in order to cope with variation in the abiotic and biotic environment, at least in the case of Chinese fir.

Furthermore, we find that the scaling exponents are all correlated with their associated normalization constants across all of the stem volume scaling relationships. Consistent with the findings of Zianis and Mencuccini^6^, Djomo *et al*.^10^, and Sileshi^28^, our analyses show a positive correlation between the numerical values of the normalization constants and scaling exponents of *D* vs. *V* (Fig. 3a). Likewise, noting that *H* = β_3_*D*^*b*/*a*^ (see Supplementary Eq. (1)), it follows that β_3_ = *H*/*D*^*b/a*^. Given that the scaling of *H* vs. *D* should shift from 1.0 to 2/3 as trees grow in size^15^,^21^,^25^, we must expect a negative relationship between β_3_ and *b*/*a*. Indeed, our data support the expectation that normalization constants significantly correlate with scaling exponents for *H* vs. *D* (Fig. 3c). Importantly, beyond the variations of scaling exponents suggested by Price *et al*.^27^, our mathematical derivation (see Eq. (9)) and empirical data illustrate that the plant fractal traits *a* and *b* influence not only scaling exponents but also normalization constants interrelating tree height, diameter, and stem volume (Fig. 4). It is reasonable therefore to argue that other key plant functional traits underlie these relationships. For example, across woody plants, wood density is a crucial variable in carbon estimation and correlates with numerous morphological, mechanical, physiological, and ecological properties of trees^46^,^47^,^48^. For example, wood density is related to the normalization constants for *M* vs. *D*^9^,^25^,^49^, but is negatively correlated to tree growth rates^47^,^50^,^51^, i.e. species with denser wood tend to have slower growth rates than species with less dense wood because dense wood may have a lower conduit fraction that reduces the rate of transpiration and photosynthesis, and thus growth in biomass. In addition, denser wood requires more mass per volume such that for the same growth in mass, a denser wood species will grow less in volume^52^. Given that the growth rate is directly proportional to plant metabolic rate^14^,^53^, wood density, constrained by plant growth, might therefore affect the scaling exponents of plant metabolism. Indeed, King *et al*.^54^ report that the scaling exponent of tree stem growth rate vs. light-interception is negatively correlated with wood density. Furthermore, wood density varies more than an order of magnitude across species^47^,^48^,^55^, which can weaken correlations among scaling exponents among biomass scaling relationships across species, because there is less variation in ecophysiological traits within species than across species. Indeed, our data reveal a stronger allometric covariation among height, diameter and stem volume relationships (i.e. *r*^2^ > 0.70) then that reported by Price *et al*.^27^ (i.e. *r*^2^ ≥ 0.27) (Fig. 2). This feature may reflect a more consistent sampling methodology for a single species with more uniform anatomical, morphological, and biomechanical properties, which would reduce the residual variation in contrast to the many differences among the many species represented in a global data set^27^.

### The prediction model

In terms of its practical application, our prediction model (Eq. (13)) was developed based on the theoretical framework of the covariation of scaling exponents and the correlation between scaling exponents and constants in stem volume scaling relationships (Supplementary Eqs (8–11)). A considerable number of studies have attempted to develop a general predictive model for biomass estimation. For example, using the small tree sampling scheme (SSS), Zianis and Mencuccini^7^ reported that the mean absolute percentage error (MAPE) of aboveground biomass estimation in 10 different studies ranged from 7.43% to 31.59%, with a mean value of 14.83%. Furthermore, using biomass-diameter-height regression models, the MAPE values of biomass estimation in tropical forests reported by Chave *et al*.^47^ ranged between 9.4% to 12.2%, with a mean value of 10.7% (recalculated by Ref. 27). Likewise, using the site-specific *H* vs. *D* scaling relationship in our field sites, MAPE across the 12 model calibrated sites listed in Supplementary Table S1 was 10.5%, and more than 57% of all MAPE values were less than 10% (Fig. 6). Consequently, our data show that the implementation of the prediction model developed here can result in very accurate predictions for stem volume. Furthermore, the data required for determining a site-specific *H* vs. *D* scaling relationship are easy to collect, requiring only height and diameter measurements. Therefore, the prediction model reported here provides a useful non-destructive tool for predicting stem volume (and biomass) based on the site-specific height vs. diameter relationship. However, it must be noted that our prediction model was developed using data drawn from the monospecific plantations having small or little variations in density and age. Another concern is the statistical limitations to fitting log-log linear power functions to *H* vs. *D* relationships, which the reductionist model is contingent upon. This latter problem is likely exacerbated when a strict linearity does not true (i.e., under less uniform conditions and a wider range of tree ages, height-diameter relationships become more complex).

## Conclusions

Our results reveal important departures from the general scaling relationships predicted for allometrically ideal plants, and show that volume scaling relationships vary significantly even for a single species. Nevertheless, a modified stem volume scaling model governed by the covariation of the numerical values of scaling exponents and normalization constants for whole-plant morphology and stem volume is shown to have remarkable predictive properties. Furthermore, the theory and empirical data present in the current study support the view that allometric normalization constants are influenced by plant fractal traits, and are thus directly related to scaling exponents (Fig. 3). Lastly, the covariation of scaling relationships provides an accurate non-destructive method for predicting Chinese fir stem volume relationships. Collectively, our data and our theory show that the changes in the numerical values of scaling exponents and the corresponding normalization constants attending growth reflect anatomical and ecophysiological responses to ontogenetic changes in size, and, importantly, differences in site quality and/or management practices. Our results provide strong circumstantial support for the hypothesis that plant growth adjusts hydraulic geometry and function in order to cope with ontogenetic changes in plant size and variation in abiotic environmental factors. Nevertheless, progress toward understanding the mechanisms that govern the scaling of plant form and function require additional theoretical insights regarding how and why scaling exponents and normalization constants covary within species. It also requires additional data in order to assess theoretical predictions.

## Additional Information

**How to cite this article**: Zhang, Z. *et al*. A predictive nondestructive model for the covariation of tree height, diameter, and stem volume scaling relationships. *Sci. Rep.* **6**, 31008; doi: 10.1038/srep31008 (2016).

## Supplementary Material

Supplementary Information
