# Supplementary material for: A predictive nondestructive model for the covariation of tree height, diameter, and stem volume scaling relationships
Source: Sci Rep. 2016 Aug 24;6:31008. doi: 10.1038/srep31008 (PMC4995560; doi:10.1038/srep31008)

Title: Covariation of stem volume scaling relationships: a predictive, non-destructive model for Chinese fir in Jiangxi Province, China

Zhongrui Zhang[1,2], Quanlin Zhong[1,2], Karl J. Niklas[3], Liang Cai[4], Yusheng Yang[2] and Dongliang Cheng[1,2*]

[1]*Fujian Provincial Key Laboratory of Plant Ecophysiology, Fujian Normal University, Fuzhou, Fujian Province 350007, China*

[2]*Key Laboratory of Humid Subtropical Eco-geographical Process, Ministry of Education, Fuzhou, Fujian Province 350007, China*

[3]*Section of Plant Biology, School of Integrative Plant Biology, Cornell University, Ithaca, NY 14853, USA*

[4] *College of Information Engineering, Jiangxi University of Technology, Nanchang, Jiangxi Province 330098, China*

[*]Authors for correspondence: E-mail: chengdl02@aliyun.com

Tel: +86 591 83465397, Fax: +86 591 83465397

Supplementary Information

Stem volume scaling relationships and the prediction model

Metabolic scaling theory (MST) characterizes a plant as a hierarchical and symmetrical branching network from which the scaling exponent relating plant metabolic rate and total mass *M* can be expressed as $\delta = \frac{1}{(2a+b)}$, where *a* and *b* are fractal traits[1]. At the individual plant level, diameter *D*, height *H,* and biomass *M* are related as:

$$D = \beta_1 M^{a\delta}\ ,\ H = \beta_2 M^{b\delta} \text{ and } H = \beta_3 D^{\frac{b}{a}} \quad (1)$$

Thus, the biomass-scaling relationships can be recast in terms of *a* and *b* as[2]:

$$D = \beta_1 M^{a\delta} = \beta_1 M^{\frac{1}{2+(\frac{b}{a})}} \quad (2)$$

$$H = \beta_2 M^{b\delta} = \beta_2 M^{\frac{1}{1+2/(\frac{b}{a})}} \quad (3)$$

Replacing $a\delta$ with *y*, $\frac{b}{a}$ with *x*, and $b\delta$ with *z* in Eqs. (1) - (3), we see that the scaling exponents take more transparent forms, i.e.,

$$y = \frac{1}{2+x}\ ,\ z = \frac{1}{1+\frac{2}{x}} \text{ and } y = \frac{1-z}{2} \quad (4)$$

which show the covariation among the numerical values of the scaling exponents governing whole plant biomass, height, and basal stem diameter[2].

These derivations show further that the normalization coefficients must covary as do the scaling exponents interrelating *D*, *H* and *M*. Specifically, we see that

$$\beta_1 = \left(\frac{\beta_2}{\beta_3}\right)^{\frac{a}{b}} = \left(\frac{\beta_2}{\beta_3}\right)^{\frac{1}{x}} \quad (5)$$

Thus, the numerical values of *a* and *b* affect scaling relationships not only in terms of their scaling exponents (Eq. (4)), but also their normalization constants (Eq. (5)). It

should be noted that Price et al.[2] examined the covariation of scaling exponents but not the covariation of the normalization constants.

Because the biomass-scaling relationship[2] focuses on the covariation among the numerical values of the scaling exponents governing whole plant biomass, height, and diameter, and because the actual biomass of the sampled trees in our study was not measured, we focus here on the relationships among *V*, *D*, and *H* (i.e. the volume-scaling relationships) and assume for the sake of simplicity that bulk tissue density varies significantly less among different species or plants compared to variations in *D*, *H*, or *V*.

This focus is predicated on the statistically strong relationship between *M* and *V* (owing to the relative constancy of wood density for any specific tree species) and on empirically strong evidence that the relationship between these *M* and *V* conforms to a formula that takes the form of $M = \beta_4 V^{\varphi}$, where φ is numerically related to the scaling between whole tree biomass and stem volume. Using this general formula, we see that Eqs. (2) and (3) can be rearranged as:

$$D = \beta_1 \beta_4 V^{\varphi a \delta} = \beta_5 V^{\frac{\varphi}{2+(\frac{b}{a})}} \qquad (6)$$

$$H = \beta_2 \beta_4 V^{\varphi b \delta} = \beta_6 V^{\frac{\varphi}{1+2/(\frac{b}{a})}} \qquad (7)$$

Similarly, replacing $\varphi a \delta$ with y', $\frac{b}{a}$ with x, and $\varphi b \delta$ with z' in Eqs. (2), (6) and (7), gives the modified MST model formula for stem volume as:

$$y' = \frac{\varphi}{2+x}\ ,\ z' = \frac{\varphi}{1+\frac{2}{x}}\ \text{and}\ y' = \frac{\varphi - z'}{2} \qquad (8)$$

Eq. (5) can then be recast as:

$$\beta_5 = \left(\frac{\beta_6}{\beta_3}\right)^{\frac{1}{x}} \quad \text{or} \ \log \beta_5 = \frac{\log \beta_6 - \log \beta_3}{x} \qquad (9)$$

Thus, the covariation functions of stem volume scaling exponents manifest a form similar to those of biomass scaling exponents.

In principle, covariation relationships of this form can be used to predict stem volume based on the empirical determination of diameter-height relationships, since the scaling exponent is inversely related to the constant in the *M* vs. *D* relationship[3,4,5], i.e.

$$\log\ \beta_5 = \frac{c}{y'} + d \qquad (10)$$

where *c* and *d* can be estimated via regression. If this relationship holds true for *V* vs. *D*, the normalization constant can be predicted from Eq. (10).

In summary, a prediction model for estimating tree stem volume can be established based on stem volume-scaling relationships. Specifically, the exponent for the *H* vs. *D* scaling relationship can be used to estimate the exponent for *V* vs. $D^{(\frac{1}{y'})}$ from Eq. (8) as $\frac{1}{y'} = \frac{2+x}{\varphi}$, and $\frac{1}{y'}$ can be used to estimate $\log \beta_5$ from Eq. (10):

$$V = 10^{\left[(\frac{2+x}{\varphi})\log D + \frac{c(2+x)}{\varphi} + d\right]} \qquad (11)$$

Eq. (11) might provide a particularly attractive method for estimating tree stem volume scaling relationships because data for *D* and *H* can be collected non-destructively.

References

1. West, G.B., Brown, J.H., & Enquist, B.J. A general model for the structure and allometry of plant vascular systems. *Nature* **400,** 664–667(1999).
2. Price, C.A., Enquist, B.J., & Savage, V.M. A general model for allometric covariation in botanical form and function. *Proc. Natl. Acad. Sci. USA* **104,** 13204–13209(2007).

3. Zianis, D. Predicting mean aboveground forest biomass and its associated variance. *For. Ecol. Manage.* **256,** 1400–1407(2008).
4. Zianis, D., & Mencuccini, M. On simplifying allometric analyses of forest biomass. *For. Ecol. Manage.* **187,** 311–332(2004).
5. Sileshi, G.W. A critical review of forest biomass estimation models, common mistakes and corrective measures. *For. Ecol. Manage.* **329,** 237–254(2014).

Table S1. Summary of climate and *Cunninghamia lanceolata* size variables at 24 sites in Jiangxi Province.

Site	Mean annual precipitation (mm)	Mean annual temperature (°C)	*H/D*	Diameter range (cm)	height range (m)	Stem volume range (m^3)
Anfu[*]	1553	17.7	0.7559	10.70-25.10	10.6-17.6	0.0591-0.395
Anyuan[*]	1640	18.7	0.7911	5.70-30.20	5.13-22.6	0.00980-0.605
Boyang[*]	1608	17.3	0.7812	6.00-19.90	5.4-15.8	0.00901-0.236
Congyi[*]	1615	17.8	0.8055	6.20-26.50	5.5-20.3	0.0112-0.453
Dexing[*]	1901	17.0	0.8600	5.30-23.60	5.8-19.6	0.00862-0.386
Guixi[*]	1832	18.2	0.7266	16.60-22.80	11.8-17.1	0.128-0.335
Hukou[*]	1442	17.4	0.7808	8.50-23.40	7.4-16.9	0.0249-0.388
Jingdezhen[*]	1650	17.4	0.7439	6.80-25.00	6.7-18.9	0.0146-0.417
Lichuan[*]	1749	17.9	0.7452	15.20-29.30	11.3-21.7	0.124-0.735
Nancheng[*]	1642	17.8	0.7906	10.20-26.60	8.3-20	0.0406-0.520
Pengze[*]	1421	16.6	0.7995	6.30-20.50	5.5-15.6	0.00992-0.247
Ruichang[*]	1700	17.5	0.6852	5.40-24.00	4.4-15.7	0.00782-0.327
Ruijin[†]	1710	18.9	0.7966	6.10-25.70	5.4-22.6	0.0131-0.547
Wuning[†]	1458	16.5	0.7849	6.80-23.60	7.4-16.3	0.0132-0.345
Wuyuan[†]	1962	16.8	0.8273	5.20-25.60	6.2-18.1	0.00895-0.414
Xingfeng[†]	1510	19.5	0.8303	10.20-21.00	8.5-16.1	0.0364-0.247
Xingguo[†]	1516	18.8	0.8162	5.20-17.30	5.8-12.6	0.00735-0.136
Xinyu[†]	1590	18.1	0.7363	16.80-25.80	11.9-19.9	0.128-0.481
Xiushui[†]	1580	16.5	0.7960	6.10-28.50	5.7-15.8	0.00967-0.482
Yanshan[†]	1730	17.9	0.6931	11.00-25.00	8.8-17	0.0529-0.379
Yichun[†]	1624	16.9	0.8185	7.80-25.00	8.9-20	0.0261-0.470
Yihuang[†]	1749	17.3	0.6767	10.30-24.20	6.3-18.2	0.0309-0.399
Yongfeng[†]	1627	18.0	0.7376	6.40-29.40	6.9-19.9	0.0132-0.646
Yongxin[†]	1550	18.2	0.7800	18.90-29.00	12.80-19.10	0.204-0.555

[*]sites used to develop the reductionist model. [†]sites used to calibrate the reductionist model.

Table S2. Summary of the reduced major axis (RMA) regression parameters (scaling exponents and normalization constants, respectively) for volume scaling relationships of height *H*, diameter *D*, and stem volume *V* for *Cunninghamia lanceolata* at 24 sites in Jiangxi Province.

Site	*n*	r^2	scaling exponents of *D* vs. *V*	normalization constants of *D* vs. *V*	r^2	scaling exponents *H* vs. *V*	*Y* - intercepts of *H* vs. *V*	r^2	scaling exponents of *H* vs. *D*	normalization constants of *H* vs. *D*
Anfu	13	0.969	0.427	1.584	0.747	0.277	1.351	0.591	0.650	0.322
Anyuan	160	0.967	0.383	1.543	0.895	0.356	1.408	0.781	0.930	-0.0268
Boyang	45	0.968	0.369	1.536	0.910	0.357	1.414	0.807	0.969	-0.0740
Congyi	79	0.971	0.387	1.542	0.894	0.324	1.391	0.792	0.838	0.0998
Dexing	125	0.966	0.396	1.548	0.885	0.296	1.383	0.774	0.746	0.229
Guixi	14	0.799	0.323	1.512	0.811	0.513	1.487	0.400	1.589	-0.915
Hukou	6	0.986	0.384	1.549	0.972	0.299	1.350	0.934	0.778	0.146
Jindezhen	89	0.942	0.383	1.548	0.808	0.358	1.397	0.622	0.937	-0.0534
Lichuan	22	0.969	0.349	1.520	0.920	0.372	1.404	0.820	1.065	-0.213
Nancheng	19	0.942	0.378	1.532	0.879	0.339	1.397	0.707	0.897	0.0230
Pengze	20	0.985	0.366	1.525	0.960	0.344	1.402	0.910	0.941	-0.0336
Ruichang	25	0.982	0.403	1.590	0.914	0.321	1.329	0.847	0.796	0.0628
Ruijin	185	0.960	0.376	1.535	0.863	0.373	1.429	0.727	0.993	-0.0941
Wuning	72	0.974	0.407	1.571	0.886	0.275	1.342	0.798	0.676	0.279
Wuyuan	106	0.981	0.397	1.552	0.893	0.300	1.372	0.826	0.757	0.197
Xingfeng	18	0.961	0.367	1.519	0.910	0.342	1.415	0.806	0.931	0.000228
Xingguo	47	0.968	0.393	1.563	0.875	0.296	1.345	0.765	0.753	0.168
Xinyu	14	0.931	0.345	1.518	0.879	0.390	1.407	0.806	1.129	-0.307
Xiushui	67	0.977	0.411	1.577	0.852	0.248	1.303	0.778	0.603	0.353
Yanshan	26	0.949	0.383	1.565	0.907	0.377	1.401	0.756	0.989	-0.147
Yichun	21	0.972	0.402	1.541	0.816	0.265	1.356	0.694	0.661	0.337
Yihuang	12	0.983	0.332	1.524	0.954	0.414	1.421	0.896	1.248	-0.480
Yongfeng	79	0.963	0.392	1.558	0.789	0.347	1.383	0.668	0.885	0.00396
Yongxin	9	0.941	0.363	1.533	0.907	0.406	1.395	0.735	1.119	-0.321

Figure S1. Locations of the 24 plantations of *Cunninghamia lanceolata* in Jiangxi Province examined in this study. Maps were generated using ArcGIS 10.0 (www.esri.com/software/arcgis)

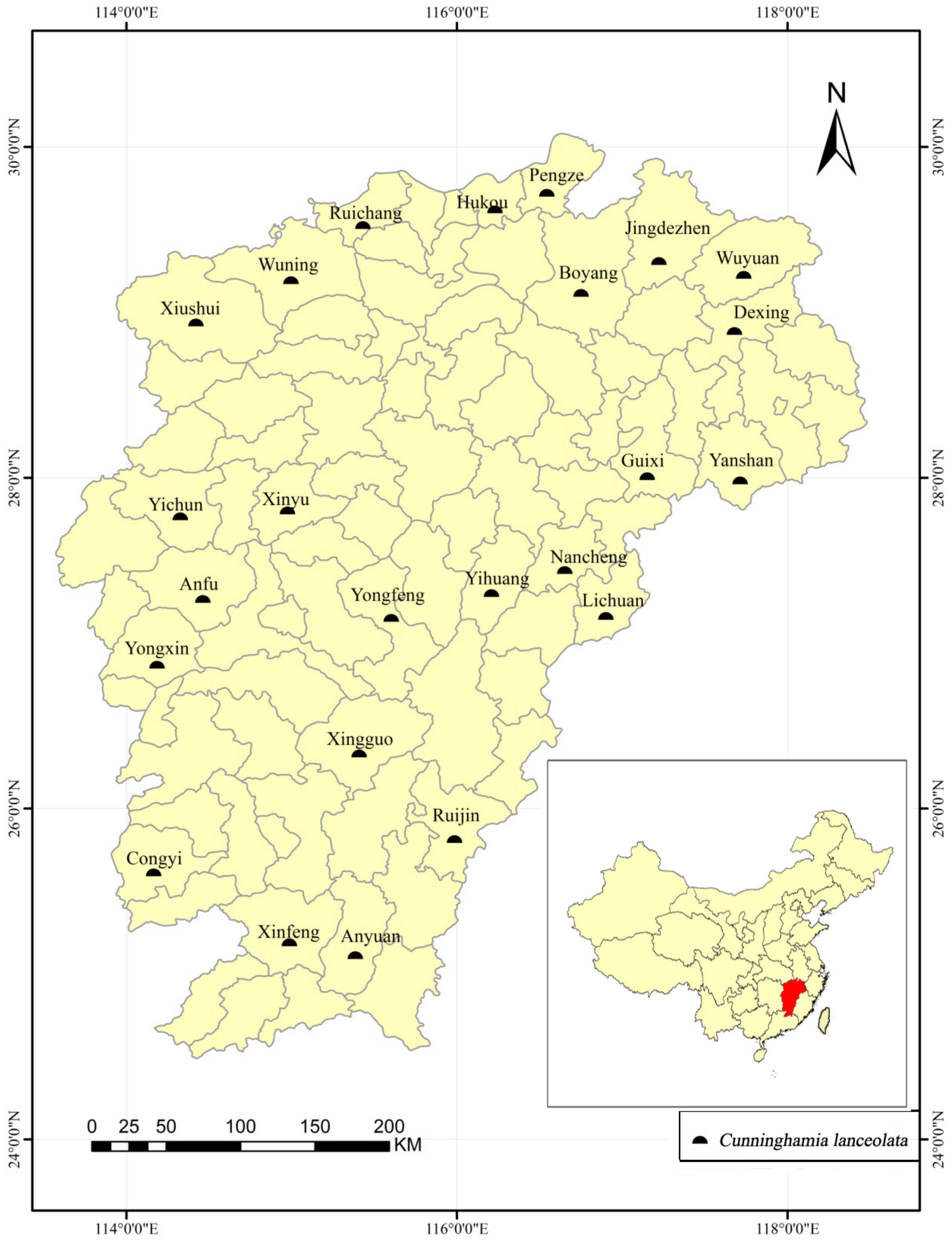

Supplement: Supplementary Information [file srep31008-s1.pdf]
